# Midline preperitoneal repair for incarcerated and strangulated femoral hernia

**DOI:** 10.1007/s10029-018-1848-3

**Published:** 2018-11-17

**Authors:** X.-M. Jiang, R.-X. Sun, W.-H. Huang, J.-P. Yu

**Affiliations:** Department of General Surgery, Jinshan Hospital of Fudan University, No. 1508 Longhang Road, Jinshan District, Shanghai, 201508 China

**Keywords:** Femoral hernia, Preperitoneal repair, Incarcerated, Strangulated, Midline incision, Tension-free repair

## Abstract

**Objective:**

Femoral hernias constantly present as incarceration or strangulation and require emergency surgery. Incarcerated and strangulated femoral hernia repair remains challenging and controversial. The aim of our study was to analyze the efficacy of preperitoneal tension-free hernioplasty via lower abdominal midline incision for incarcerated and strangulated femoral hernia.

**Methods:**

Data of 47 patients who underwent emergency surgery for incarcerated or strangulated femoral hernias from January 2009 to December 2017 were retrospectively analyzed. According to the surgical incisions, they were divided into two groups: the observation group (21 cases) had a lower abdominal midline incision, and the control group (26 cases) had a traditional inguinal incision. General data of patients, intraoperative findings, operative time and postoperative complications were compared.

**Results:**

Patient characteristics showed that the two groups were comparable.15 cases (31.9%) underwent intestinal resection, and 32 cases (68.1%) underwent first-stage tension-free repair in total. The rate of first-stage tension-free hernioplasty was significantly higher in the observation group (18/21, 85.7% vs 14/26 53.8%, *P* = 0.020). No additional incision was required in the observation group, while six cases of the control group (23.1%) had an additional incision for intestinal resection and anastomosis (*P* = 0.026). Mean operative time (53.6 ± 24.7 min vs 77.9 ± 36.5 min, *P* = 0.012) and the length of hospital stay (6.3 ± 4.2 days vs 10.3 ± 6.9 days, *P* = 0.020) were significantly shorter in the observation group. The time of return to normal physical activity resulted significantly reduced compared to the control group (9.2 ± 4.1 days vs 13.3 ± 6.6 days, *P* = 0.017). The total incidence of postoperative complication (including chronic pain, foreign body sensation, hernia recurrence, wound infection and seroma/hematomas) in the observation group was lower (14.3% vs 42.3% *P* = 0.037). There were two recurrences in the control group. No mesh-related infection and no mortalities in two groups.

**Conclusions:**

Midline preperitoneal approach for incarcerated and strangulated femoral hernia is a convenient and effective technique. It can improve the rate of first-stage tension-free repair of incarcerated femoral hernia and allow intestinal resection through the same incision, and with lower rate of postoperative complications.

## Introduction

Femoral hernia is relatively rare, but it often presents incarceration and intestinal necrosis. About 30.4–45.9% of femoral hernias [1–4] presenting commonly as a surgical emergency with high rates of exploratory laparotomy and intestinal resection [5]. Intestinal resections may be required in 9.3–33.7% [4, 6–9] of incarcerated femoral hernias due to necrosis, and mortality rates can be as high as 4.9% [10].

There are different surgical repair techniques and incisions for incarcerated and strangulated femoral hernia. Classical McVay repair is commonly used and recommended by some guidelines. The traditional approach via an inguinal incision for incarcerated femoral hernia has been widely adopted, and 10.5–27.8% patients [1, 11–13] who required exploratory laparotomy through an additional abdominal incision developed some type of complications. Exploratory laparotomy in strangulated hernia surgery might cause postoperative complications, besides 50% of midline laparotomies were performed without any intestinal resection [13]. In conventional repair, high tension is associated with acute pain and recurrence [14]. Mesh repair of incarcerated or strangulated hernias is controversial, especially when intestinal resection is required [7]. Mesh plug repair is simple and effective, but it is not favorable for potentially unidentified coincidental direct and indirect hernias [14], and the persistence of postoperative discomfort in some patients [15]. The preperitoneal approach repairs have some advantages, such as lower postoperative complication rate, and earlier return to full activity [16]. Because of minimally invasive and cosmetic advantages, laparoscopic preperitoneal hernia repair was recommended by some guidelines for inguinal hernia repair when the surgeon is experienced [17], but not in an emergency. For higher treatment costs and longer learning curve, laparoscopic extraperitoneal repair has not been widely applied in incarcerated and strangulated femoral hernias.

Open preperitoneal mesh repair via lower abdominal midline incision may be a standard approach for incarcerated or strangulated femoral hernia. It enables immediate release of intestine obstruction and reduces damage or bowel perforation. Extraperitoneal mesh repair can be accomplished through the same incision. This approach can obtain a good exposure and an easy access if intestine resection is necessary. Several studies have analyzed preperitoneal repair via lower abdominal midline incision for intestinal resection in inguinal hernias but not specifically for incarcerated and strangulated femoral hernia [18, 19]. We conducted a retrospective analysis to evaluate the advantages of preperitoneal tension-free hernia repair via lower abdominal midline incision for incarcerated or strangulated femoral hernia.

## Methods

A retrospective analysis was performed on data collected prospectively over a 9-year interval from January 2009 to December 2017. 47 adult patients underwent emergency treatment for incarcerated or strangulated femoral hernia at Jinshan Hospital of Fudan University in Shanghai, China. Preoperative workup including blood analysis, an electrocardiogram, chest radiography, abdominal computed tomography, and risk assessment of anesthesia. There was no recurrent or bilateral femoral hernia. Attempt for the manual reduction of femoral hernias was banned in our hospital. Contents of hernia sac back to the abdominal cavity before surgery were excluded. 21cases (observation group) had emergency operation through a lower abdominal midline incision and 26 cases (control group) via an inguinal incision. The protocol was approved by the Ethics Committee of Jinshan Hospital, Fudan University. General data of patients (Table 1), need for intestinal resection, operative time, surgical approach, length of hospital stay, postoperative complications and recurrence rates were compared.

**Table 1:** Demographic data and clinical characteristics of the patients

Parameter	Midline incision group (*N* = 21)	Inguinal incision (control group) (*N* = 26)	*P*
Gender (male/female) *n*	3/18	7/19	0.488
Age (years)	73.3 ± 10.8	76.6 ± 10.5	0.305
BMI	21.4 ± 1.5	22.4 ± 2.2	0.103
Duration of incarceration (h)	9.9 ± 4.8	10.8 ± 5.8	0.536
Side of hernia (right/left) *n*	13/8	14/12	0.579
ASA score *n* (%)			
I	3 (14.3%)	5 (19.2%)	0.618
II	9 (42.9%)	7 (26.9%)	
III	8 (38.1%)	11 (42.3%)	
IV	1 (4.8%)	3 (11.5%)	
Contents of hernia sac *n* (%)			
Intestine	16 (76.2%)	18 (69.2%)	0.690
Omentum	4 (19.1%)	5 (19.2%)	
Others	1 (4.8%)	3 (11.5%)	
Intestinal resection *n* (%)	6 (28.6%)	9 (34.6%)	0.899
Duration of follow-up (month)	52.7 ± 30.2	60.1 ± 34.7	0.443

*ASA* American Society of Anesthesiology score, *BMI* body mass index

Surgeries were performed under spinal or general anesthesia by the same surgeon team. To maintain an empty bladder, all patients were inserted a urinary catheter which was removed at the end of the operation. Prophylactic antibiotics were administered in all cases, and a postoperative antibiotic treatment was performed 2–5 days. The antibiotics used were cefuroxime or alternatively levofloxacin.

Midline incision group (observation group): the procedure was performed via lower abdominal midline incision (4–6 cm), which lower edge was attached to the superior margin of the pubis. The skin, the subcutaneous tissue and the anterior sheath of rectus abdominis were incised, and then rectus abdominis muscles were separated along the midline. Transverse fascia was incised, then the extraperitoneal space between the peritoneum and transversalis fascia was exposed. The dissection of preperitoneal space was same with totally extraperitoneal repair (TEP). This approach can observe the hernia sac directly. If the hernia could not be reduced by pulling the hernia sac, then the inguinal ligament should be incised to enlarge the femoral ring for reduction. Inversion of the sac and visual inspection of the contents through transparent peritoneum were performed if necessary. The mesh (11 cm × 14 cm, Bard USA) was placed in preperitoneal space, covering all the facial defects (Hesselbach’s triangle, indirect ring, femoral triangle, and obturator ring) in the groin to prevent recurrence and other hernias. The mesh was fixed to sheath of rectus abdominis to prevent wrinkling and displacement. Whenever resection of nonviable intestine was to be undertaken, the operative field was protected from contamination with saline soaked pads. Intestinal resection was not considered as a contraindication for prosthetic repair. In cases of severe generalized peritonitis, or evidence of spilling intestinal contents into the surgical field, we preferred hernia sac ligation or resection and suture closure of the femoral canal.

Inguinal incision group (control group): the procedure is performed through an oblique incision above the lateral one-third of the inguinal ligament or transverse incision to achieve better cosmetic result. The hernia sac was routinely opened and the contents were examined. The surgeon individually decided which surgical technique to use according to the intra-operative observations. Repair methods including Modified Kugel mesh, mesh plug method and tissue repair (McVay method, only hernia sac ligation, hernia sac resection) were chosen based on intra-operative findings.

Patient information including sex, age, BMI, personal health history, side of hernia, duration of incarceration, contents of hernia sac, the surgical technique used, the time of operation, the length of hospital stay, perioperative complications (wound infection, acute pain, seroma/hematomas), the main long-term complication (recurrence, chronic pain, foreign body sensation) and mortality were recorded and analyzed. Follow-up visit was conducted 1 week after surgery for routine physical examination. Then 3 months after surgery, patients were adopted with routine out-patient clinic, returning visit or telephone follow-up and assessed for postoperative complications.

Statistical analysis was performed using SPSS version 22 (IBM, USA). Data were presented as mean ± standard error of the mean. Univariate comparisons were made by unpaired Student’s *t* test and Chi-square analysis. Fisher’s exact test was used when any expected cell value in a 2 × 2 table was less than 5. *P* < 0.05 was regarded as significant.

## Results

From Jan 2009 until Oct 2017, 47 patients diagnosed as incarcerated or strangulated femoral hernias were treated in the Jinshan Hospital of Fudan University. The average age was 75.1 ± 10.7 years, 37 of them (78.7%) were female. 15 cases (31.9%) underwent intestinal insection. 32 cases (68.1%) underwent first-stage tension-free repair in total. There were 21 patients in the observation group, and female/male ratio was 18/3. The control group was composed of 26 patients (female/male ratio 19/7). Patient information given in Table 1 showed that the two groups were comparable. The differences in follow-up duration and bowel resection were statistically insignificant.

**Table 2:** Operative outcome

Outcome	Midline incision group (*N* = 21)	Inguinal incision (control group) (*N* = 26)	*P*
Tension-free repair *n* (%)	18 (85.7%)	14 (53.8%)	0.020
Wound infection *n* (%)	1 (4.8%)	4 (15.4%)	0.485
Seroma/hematomas *n* (%)	2 (9.5%)	3 (11.5%)	1.000
Laparatomy by an additional incision *n* (%)	0	6 (23.1%)	0.026
Acute pain (require medication) *n* (%)	8 (38.1%)	15 (57.7%)	0.062
Operation time (min)	53.6 ± 24.7	77.9 ± 36.5	0.012
Postoperative duration of stay (days)	6.3 ± 4.2	10.3 ± 6.9	0.020
Time to return to full activity (days)	9.2 ± 4.1	13.3 ± 6.6	0.017
Sensation of foreign body *n* (%)	0	1 (3.8%)	1.000
Postoperative chronic pain *n* (%)	0	1 (3.8%)	1.000
Recurrence *n* (%)	0	2 (7.7%)	0.567
Postoperative complications (total) *n* (%)	3 (14.3%)	11 (42.3%)	0.037

Total including wound infection, seroma/hematomas, foreign body sensation, chronic pain, and hernia recurrence

The surgical methods used for the observation group were preperitoneal tension-free hernia repair in 18 cases (85.7%), and conventional repair (suture closure of the femoral canal) in three patients (Table 2). The follow-up time ranged from 6 to 100 months with a mean of 52.7 ± 30.2 months, there were no recurrences in midline incision group. In the control group,14 patients (53.8%) underwent first-stage tension-free repair including the modified Kugel patch method in 4 (15.4%) and mesh plug method in 10 (38.5%). Ten patients underwent conventional repair including McVay method in 6 (23.1%), suture closure of the femoral ring in 2 (7.7%), only hernia sac ligation or resection in 2 (7.7%). The follow-up time ranged from 6 to 97 months with a mean of 60.1 ± 34.7 months. Inguinal hernia recurrences developed in two of conventional repair cases who underwent reoperation by mesh plug repair.

In the observation group (21cases), intestinal resection was performed in six patients (28.6%). 18 patients (18/21, 85.7%) underwent preperitoneal tension-free repair, one case had wound infection, and two cases had hematoma or seroma. In control group (26 cases), intestinal resection was performed in nine patients (34.6%), six of them underwent intestinal resection and anastomosis by an additional incision, while no additional incision was required in the observation group (*P* = 0.026). 14 patients (14/26, 53.8%) underwent first-stage tension-free repair. The rate of herniorrhaphy was significantly higher in the observation group (85.7% vs 53.8%, *P* = 0.020). There were no significant differences between the two groups in perioperative acute pain (38.1% vs 57.7%, *P* = 0.062) and wound infection (4.8% vs 15.4% *P* = 0.485). The total incidence of postoperative complication (including chronic pain, foreign body sensation, hernia recurrence, wound infection and seroma/hematomas) in the observation group was lower than that in the control group (3/21 14.3% vs 11/26 42.3% *P* = 0.037). Mean operative time (53.6 ± 24.7 min vs 77.9 ± 36.5 min, *P* = 0.012) and the length of stay (6.3 ± 4.2 days vs 10.3 ± 6.9 days, *P* = 0.020) were significantly shorter for the observation group. The time to return to full activity was significantly reduced in the observation group, compared to the control group (9.2 ± 4.1 days vs 13.3 ± 6.6 days, *P* = 0.017). The hematoma and wound infection were managed conservatively, and no mesh-related infection was seen. There were no mortalities in either group.

## Discussion

Incarcerated or strangulated femoral hernia is a serious surgical emergency, as it leads to high morbidity and mortality rate [10]. Therefore, regardless of the site and size, emergency surgery is usually the first choice for incarcerated or strangulated femoral hernia. The aim of emergent surgery of incarcerated or strangulated hernia may be to release intestinal obstruction quickly, debride necrotic tissue, and repair the hernia to reduce morbidity and mortality. In open surgery via inguinal incision, intestinal resections and anastomosis through the sac contribute to operation difficulties, local tissue damage and contamination for strangulated femoral hernia. Patients who required exploratory laparotomy by an additional incision would develop some type of complications. Surgical approach to cure incarcerated or strangulated femoral hernia remains controversial. Surgical approach including incision selection and synthetic mesh repair remains challenging and controversial. First-stage tension-free hernioplasty in cases of strangulated hernia was debated due to the potential risk of infection [20, 21]. Incarcerated or strangulated hernia was considered as a contraindication for mesh repair. A large proportion of incarcerated hernias need second-stage tension-free hernioplasty, because mesh hernioplasty has the advantage of lower rate of recurrence [22]. If protecting the surgical field from intestinal contents, the presence of intestinal ischemia or necrosis cannot be considered a contraindication for mesh repair [20]. Preperitoneal hernioplasty may be safe with a relatively lower risk of morbidity if the surgical field was not contaminated by intestinal contents [22]. Laparoscopic approach is superior to the open approach in minimizing chronic pain and numbness with a quicker return to normal activities [23]. For incarcerated or strangulated hernias, laparoscopic approach is technically more difficult than open repair [24, 25]. Preperitoneal hernia repair via lower abdominal midline incision for incarcerated femoral hernia may be the optimal approach, and it has the benefit of mesh in extraperitoneal space without disadvantages of laparoscopic repair, such as the long learning curve and iatrogenic injury.

Midline incision for femoral hernia repair was first described by Annandale in 1876, then it was improved and modified by Nyhus and Stoppa. Midline preperitoneal repair allows us to completely close the preperitoneal space between the peritoneum and transversalis fascia, and then to incise peritoneum for exploratory laparotomy. Meanwhile, the surgical area was protected by saline-soaked pad to minimize potential contamination, so that the extra-peritoneal mesh was away from abdominal effusion and intestinal contents. Over the past 9 years, there were three cases of intestinal resections and anastomosis and four cases of partial omentum resection in whom tension-free herniorrhaphy was used, and there were no severe complications or mesh-related infections in the observation group. We found that preperitoneal herniarrhaphy was effective and feasible, as long as neither serious peritonitis nor intestinal perforation. In our study, the rate of first-stage tension-free herniorrhaphy was higher in the observation group (85.7% vs 53.8%, *P* = 0.020), with a lower incidence of postoperative complication (14.3% vs 42.3% *P* = 0.037). Without increasing perioperative acute pain and wound infection, midline preperitoneal repair reduced operative time (53.6 ± 24.7 min vs 77.9 ± 36.5 min, *P* = 0.012) and the length of hospital stay (6.3 ± 4.2 days vs 10.3 ± 6.9 days, *P* = 0.020), and helped for a quicker return to full activity (9.2 ± 4.1 days vs 13.3 ± 6.6 days, *P* = 0.017). For incarcerated or strangulated femoral hernias, the inguinal ligament can be incised to enlarge the femoral ring for reduction. This incision should be performed under direct vision to keep electrotome heat away from femoral artery or vein and the contents of the hernia. Total familiarity with the preperitoneal space anatomy and the sites of releasing incision are fundamental to undertaking this approach. Since laparoscopic surgery either transabdominal preperitoneal (TAPP) or totally extraperitoneal (TEP) has been widely used non-emergency repair, it was not difficult to understand the anatomy of the extraperitoneal space. We believed with the present observation that preperitoneal hernia repair via lower abdominal midline incision applied here would justify its presence in the domain of incarcerated or strangulated femoral hernia repair.

Midline preperitoneal repair could be the best solution for incarcerated or strangulated femoral hernia. (1) Dissection through an open incision is easy to release strangulation, and structures are widely visible. Extraperitoneal mesh repair and intestinal resection can be accomplished through the same incision. When femoral hernia is wrongly diagnosed as the cause of an “acute abdomen” this approach allows adequate examination of the peritoneal contents, and no additional incision is required. (2) The dissection of preperitoneal space is away from the inferior epigastric vessels and the triangle of doom. Chronic pain was reduced by minimizing the risk of injury to the iliohypogastric nerve, ilioinguinal nerve and genitofemoral nerve by posterior dissection [26]. This approach enables the positioning of a wide mesh and to cover all the facial defects (Hesselbach’s triangle, indirect ring, femoral triangle, and obturator ring) in the groin. Thus, the risk of recurrence and sensation of foreign body would be reduced. (3) Preperitoneal repair and then exploratory laparotomy would decrease the risk of contamination from intestinal necrosis and perforation. It can improve the rate of first-stage tension-free repair in incarcerated or strangulated femoral hernia with a lower complication rate. (4) The preperitoneal approach could discover other hernia not detected on preoperative physical examination and avoid the adhesions by previous inguinal surgery [11]. The cosmetic result is favorable to repair bilateral hernias. (5) Open preperitoneal hernia repair is easier to learn than laparoscopic approach. The learning curve is shorter than laparoscopic repair. Midline preperitoneal repair is a suitable technique to repair hernia in case totally extraperitoneal (TEP) repair is unsuccessful.

Midline preperitoneal repair is different from conventional repair via inguinal incision. Preperitoneal anatomy is not an obstacle and this approach could provide a more thorough understanding of the preperitoneal area, as proposed by Hamilton et al. [27]. It should be noted that in some cases with long duration of hernia or previous pelvic and lower abdominal operation (radical prostatectomy, cystectomy, lower anterior resection of the rectum, gynecological operations, and ascites), there might be severe surrounding adhesions that cannot be dissected by simply opening the hernia sac. In this scenario, conventional repair should be considered. Obese or muscular cases were not recommended because of the difficulty of preperitoneal space dissection.

There were some limitations to this study. (1) It was a single-center retrospective research with no randomization and blinding. The relative scarcity of femoral hernias makes randomized controlled clinical study difficult. (2) 47 patients in the study were Chinese. Differences in Asian race with a relatively lower BMI may have affected the results. The small sample size may have bias. (3) Laparoscopic approach has been reported by some studies in emergency herniorrhaphy, but they are mostly case reports of strangulated femoral hernias. TEP or TAPP has not been adopted as the standard procedure and not compared in this study. Despite these limitations, our study has provided evidence that preperitoneal hernia repair via lower abdominal midline incision may be a better surgical approach for incarcerated or strangulated femoral hernia. As for whether the approach can be adopted as a standard procedure, it needs to be studied further, ideally with larger multicenter randomized controlled trials.

## Conclusion

Midline preperitoneal repair for incarcerated or strangulated femoral hernia is a convenient and effective technique. It may improve the rate of first-stage tension-free repair of incarcerated femoral hernia and allow intestinal resection through the same incision, and with lower rate of postoperative complications. It is suitable for the clinical application and can be adopted as a valid alternative in the treatment of incarcerated and strangulated femoral hernias.
